# Review of invasive urodynamics and progress towards non-invasive measurements in the assessment of bladder outlet obstruction

**DOI:** 10.4103/0970-1591.45544

**Published:** 2009

**Authors:** C. J. Griffiths, R. S. Pickard

**Affiliations:** Department of Medical Physics, Freeman Hospital, Newcastle University, Newcastle upon Tyne, UK; 1 School of Surgical and Reproductive Sciences, Newcastle University, Newcastle upon Tyne, UK

**Keywords:** Bladder, bladder neck obstruction, nomograms, non-invasive, urodynamics

## Abstract

**Objective:**

This article defines the need for objective measurements to help diagnose the cause of lower urinary tract symptoms (LUTS). It describes the conventional techniques available, mainly invasive, and then summarizes the emerging range of non-invasive measurement techniques.

**Methods:**

This is a narrative review derived form the clinical and scientific knowledge of the authors together with consideration of selected literature.

**Results:**

Consideration of measured bladder pressure urinary flow rate during voiding in an invasive pressure flow study is considered the gold standard for categorization of bladder outlet obstruction (BOO). The diagnosis is currently made by plotting the detrusor pressure at maximum flow (p_detQmax_) and maximum flow rate (Q_max_) on the nomogram approved by the International Continence Society. This plot will categorize the void as obstructed, equivocal or unobstructed. The invasive and relatively complex nature of this investigation has led to a number of inventive techniques to categorize BOO either by measuring bladder pressure non-invasively or by providing a proxy measure such as bladder weight.

**Conclusion:**

Non-invasive methods of diagnosing BOO show great promise and a few have reached the stage of being commercially available. Further studies are however needed to validate the measurement technique and assess their worth in the assessment of men with LUTS.

## INTRODUCTION

To those expected to treat patients with lower urinary symptoms (LUTS), it is notorious that “The bladder is an unreliable witness…. it has a limited means of expressing its own pathology”.[1,2] In practice, this means that the underlying cause is often unclear from symptoms, even to an expert urologist. To aid diagnosis techniques have been developed to provide objective evidence of lower urinary tract dysfunction. This review will chiefly concentrate on the differentiation of bladder outlet obstruction (BOO) and detrusor underactivity in men; both of which result in similar voiding symptoms. This distinction is clinically important since the likelihood of benefit from the available treatments will depend on making a correct diagnosis.[3,4]

## CONVENTIONAL MEASUREMENTS OF BLADDER AND OUTLET FUNCTION

### Voiding diary

**Figure 1:**
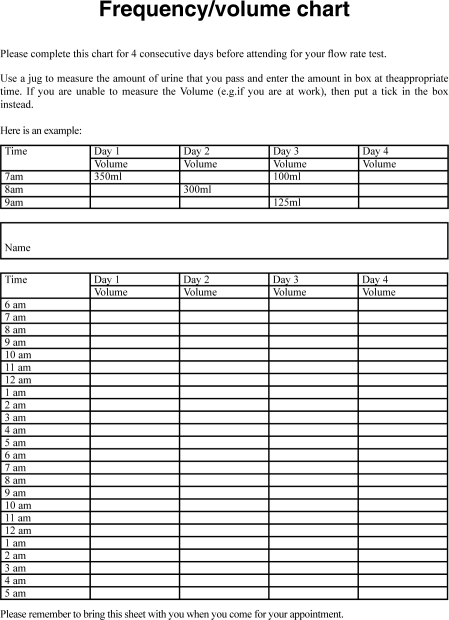
Example of a frequency volume chart which a patient may be asked to complete prior to attending a clinic

A voiding diary kept for a period of a few days provides useful additional objective information to support the patient's description of their symptoms. Most patients are able to keep such a record with the aid of a well-designed frequency/volume chart [Figure 1] and this is useful in documenting the frequency of micturition and the range of voided volume.

### Uroflowmetry

**Figure 2:**
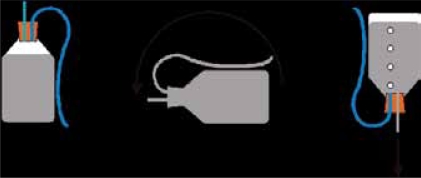
Device producing constant flow rate to calibrate a flowmeter. The vent maintains constant pressure at the level of the bung and hence constant flow rate through the outlet tube

Additional information can be obtained by asking the patient to empty their bladder into a urine flow meter. This simple measurement provides objective evidence of the normality of voiding. The methods most commonly used today are based on either a ‘load cell’ or a ‘rotating disc’. It is important to regularly calibrate such instruments and a self-contained constant flow device [Figure 2] makes this easy to do.[5]

### Pressure-flow study

**Figure 3:**
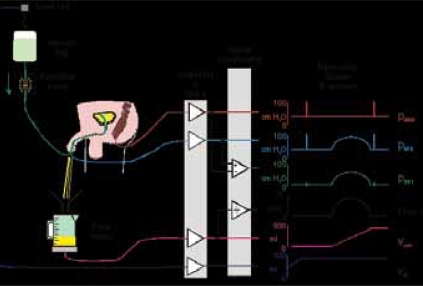
Diagram illustrating principles of recording in cystometry. Bladder pressure and rectal pressure are recorded via catheters and also subtracted, electronically, to measure the pressure generated by the bladder itself, the detrusor pressure. Voided volume is measured, typically by a load cell, and flow rate determined electronically by differentiation (If flow rate is measured directly, volume is determined by integration.) The bladder is filled by infusion through an additional catheter (or additional lumen in the bladder pressure catheter). Traditionally data was plotted on a chart recorder but is now recorded to computer disc and subsequently printed for hard copy.

A low flow rate may be due to either bladder outlet obstruction or detrusor underactivity and information about bladder pressure is required to differentiate these two abnormalities.[6] Bladder pressure is normally recorded via a fine, fluid-filled catheter passed into the bladder via the urethra with the distal end connected to an external pressure transducer. The transducer should be positioned level with the upper margin of the pubic symphysis and zeroed to atmospheric pressure. Catheters are also available with micro transducers mounted on the tip. These do not need to be leveled externally but, unlike fluid filled catheters, the pressure recorded will depend on the tip's position, height-wise, within the bladder. They are also less prone to movement artefacts and are therefore more suited to ambulatory studies (see below). The bladder transducer will also record an overall rise in abdominal pressure as well as detrusor contraction. To distinguish between these abdominal pressure is also recorded via a second transducer, usually placed in the rectum. This pressure is subtracted from the bladder pressure to derive the detrusor pressure [Figure 3]. During recording, the patient should regularly be asked to cough to check that both channels are measuring correctly with the cough pressure deflection cancelled on the subtracted trace. The International Continence Society (ICS) Good Urodynamic Practice Guide gives advice on best practice.[7] A second catheter (or double lumen catheter) permits artificial filling of the bladder, usually at 50 or 100 ml/min, though this may be reduced 10 or 20 ml/min to avoid stimulating contraction of an over-sensitive bladder.

A urodynamic study normally consists of two main phases: filling cystometry, to investigate storage, and the effect of provocative maneuvers such as coughs and stationary jogging; followed by a pressure-flow study (PFS) to investigate voiding performance.[7] Performing reliable pressure-flow studies requires an experienced operator. It is important to take a careful history beforehand to understand the patient's symptoms with the aim of reproducing these during the study. There is much advice available on the practicalities of performing pressure-flow studies[6] and ICS approved courses are available.

### Video pressure-flow studies

An important addition to pressure-flow studies is simultaneous X-ray imaging. The bladder is filled with contrast media and the video X-ray image of the bladder and outlet is recorded at strategic times during both filling and voiding. For maximum diagnostic value, the image is synchronized with the pressure-flow recordings. Commercial pressure-flow recording systems are now designed using personal computers equipped with digital video capture facilities.

### Residual bladder volume measurement

One possible consequence of voiding dysfunction is failure of the bladder to empty completely. The residual volume can be estimated with reasonable accuracy (around 10%) using ultrasonic imaging.[8] Small, hand-held scanners designed specifically for this purpose are now available (Bladderscan, Diagnostic Ultrasound, www.dxu.com).

### Ambulatory monitoring

The unnatural, and for some patients inhibitory, environment of the urodynamic clinic can be avoided by performing ambulatory urodynamics with natural filling.[9] It has been found useful for confirming overactive detrusor in patients where a conventional cystometry failed to reproduce symptoms.[10] However, overactivity can also be detected in females who are not complaining of symptoms.[11]

### Urethral pressure profile (UPP)

The pressure profile along the length of the urethra can be measured by slowly infusing a fluid through a catheter while withdrawing it through the urethra.[12] Catheter tip transducers can also be used for this purpose with claimed higher reliability.[13] The urethral pressure profile can also be recorded during micturition with the potential advantage of localizing the site of an obstruction[14] but this has not been widely adopted.

## INTERPRETATION OF URODYNAMIC MEASUREMENTS

Most men with LUTS and suspected BOO will have a flow rate measurement and some will go on to have a full invasive urodynamic investigation with the aim of answering the following questions:

- Can the bladder be filled to normal capacity without significant pressure rise due to either an overactive bladder or low compliance?
- Can the patient empty his bladder to completion with a normal flow rate and voiding pressure, without straining?

The second of these questions clearly relates to the pressure-flow study recorded during the voiding phase of the invasive investigation. The physical interpretation of pressure-flow measurements requires a model of the bladder.

### Model of the bladder and outlet

Early models assumed the bladder generated a pressure, p, which determined the flow rate, Q, emptying through a rigid tube. Laminar flow is governed by Poiseuille's equation:

R = p/Q

However, Smith noted that during voiding flow is likely to be turbulent and the relationship should be:[15]

$${\text{R = p/Q}}^{\text{2}}$$

**Figure 4:**
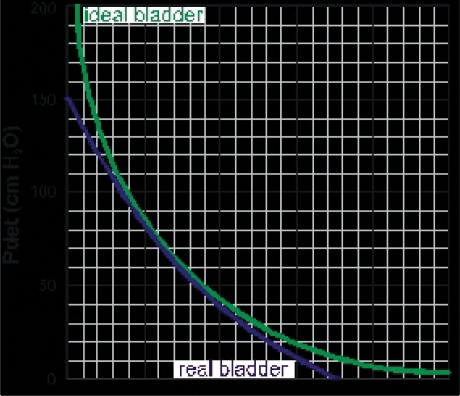
Bladder pressure – flow relationship (green trace is for constant power: PV = constant; blue trace is more realistic PV relationship derived from Hill equation).

**Figure 5:**
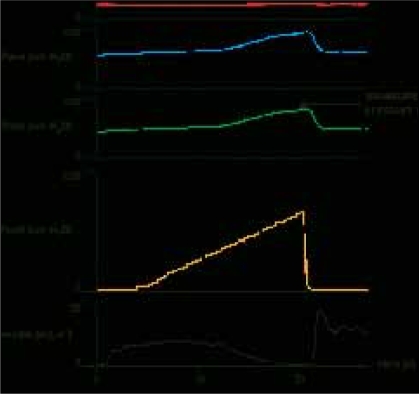
Urodynamic traces during inflation of a penile cuff (pcuff) showing rise to isovolumetric pressure as flow reduces to zero

The assumption that the pressure p is the independent variable and Q is dependent on R was brought into question by Derek Griffiths who introduced a model for the bladder and its contraction based on the Hill equation previously applied to skeletal muscle.[16,17] It can be deduced from this that the bladder operates closer to a constant source of power rather than constant pressure (From basic physics, power = pQ). The expression derived from the Hill equation includes additional constants which avoids the problem of the pressure becoming infinite at zero flow, and vice versa [Figure 4]. A consequence of this relationship is that if midstream flow is reduced to zero the pressure will rise to an isovolumetric pressure higher than at full flow for a constant bladder effort [Figure 5], corresponding to a right to upper-left movement in [Figure 4]. A good, concise summary of this theory is included as an appendix in the article by Griffiths *et al.*[18]

The idea that voiding function is a subtle interaction of bladder contraction and outlet function was developed further by Derek Griffiths and Werner Schaffer.[19,20] They introduced the important concept that the pressure required to open the urethra (p_open_) may be elevated, which they described as a compressive obstruction. To void, a significant part of the bladder pressure would be required to overcome this pressure, with only the remainder being available to accelerate the fluid and therefore create flow according to Bernoulli's theorem. On the other hand, they proposed the cross sectional area of the urethra (A) may be reduced with a low opening pressure which they described as a constrictive obstruction. This would allow all the pressure to be converted to velocity (v), but reduce the flow because of the restricted cross section area (Q = vA). From these considerations it can be shown, where ρ is the density of the fluid:[20]

$$\text{p}-{\text{p}}_{\mathrm{open}}=\rho {\left(\text{Q}/\text{A}\right)}^{2}/2$$

**Figure 6:**
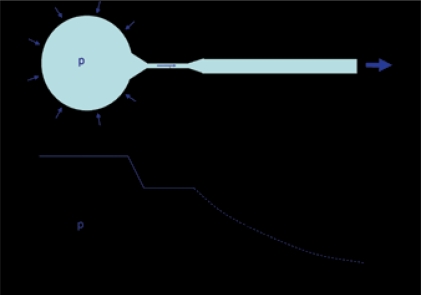
Diagram showing how the flow rate is determined by the urethral opening pressure and Bernoulli's principle at the ‘Flow Controlling Zone’

An important consequence of these ideas is that a small region of the urethra with a high opening pressure, or narrow constriction, (or both) can control the flow rate; referred to as the flow controlling zone (FCZ) [Figure 6].

### Interpretation of flow rate

The value of a flow rate test is increased if the patient has completed a frequency-volume chart beforehand to check the voided volume is typical and this should be at least 150 ml for a reliable measurement although nomograms exist to make adjustment for lower volumes.[21,22] Confirmation of an adequate volume in the bladder by ultrasonic imaging prior to the test can be helpful. Interpretation of the flow rate recording requires care. The most widely used measurement is peak flow rate, Q_max_, but often the trace will contain artefacts which make interpretation of Q_max_ difficult. If the equipment automatically measures Q_max_, this should be treated with suspicion as it can be incorrect.[7] In men Q_max_ >15 ml/s is considered normal and Q_max_ <10 ml/s is considered reduced, possibly suggestive of outlet obstruction or a weak bladder. Flow rates between these values are more difficult to interpret. This is because there is a large overlap of the distributions of Q_max_ for normal patients and patients with voiding abnormalities which limits the sensitivity and specificity that can be achieved with flow rate measurement alone. Some urologists advocate using the best of multiple measurements to improve predictive accuracy[23] but this is at the expense of sensitivity.[24]

The shape of the flow rate curve is also useful. A normal void is typically ‘bell’ shaped whereas an obstructed patient has a more extended shape with a gradual tail off, with Q_max_ occurring proportionally much earlier in the voiding cycle. Intermittent flow may be suggestive of outlet obstruction but can be associated with other conditions such as a weak bladder, straining, or poorly coordinated bladder contraction, and outlet relaxation. A flattened plateau shape to the flow rate trace may suggest urethral constriction.

Further interpretation of flow rate has been proposed[25,26] but the consensus of opinion is that additional information is required for a full diagnosis. A full invasive urodynamic study including measurement of voiding pressure provides this information.

### Interpretation of pressure-flow study

Depending on their symptoms and flow rate study, many patients will go on to have an invasive study. Diagnosis of BOO or detrusor underactivity depends on the interpretation of the pressure-flow study recorded during the voiding phase. A number of tools have been developed to help with this task, based on the models of the lower urinary tract described above.

**Figure 7:**
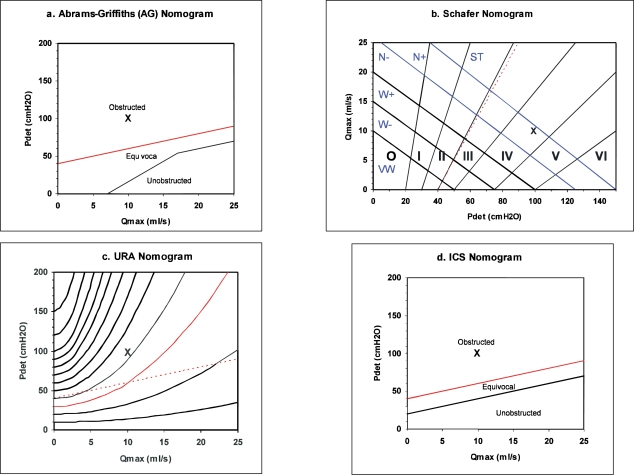
Different nomograms that have been proposed to categorize BOO. The red lines on ‘a’ and ‘d’ separating the obstructed region are identical. The equivalent line is shown as a dotted red line on ‘b’ and ‘c’ and is almost identical to the line separating grade II and grade III on the Schäfer nomogram ‘b’. X is a patient with Q_max_ of 10 ml/s and pdet.Q_max_ of 100 cmH_2_O categorized throughout as obstructed.

Paul Abrams and Derek Griffiths proposed that a continuous plot of detrusor pressure versus flow rate for the voiding period would enable diagnosis of obstruction and went on to demonstrate that a single point plot of detrusor pressure (p_det.Qmax_) at the time of maximum flow rate (Q_max_) on an XY graph could help diagnose patients.[27] They divided the graph into three regions and depending in which an individual patient was situated, they were diagnosed as ‘obstructed’, ‘equivocal’ or ‘unobstructed’ [Figure 7a]. This nomogram is commonly referred to as the ‘AG nomogram’. Patients falling into the ‘equivocal’ region can be further diagnosed depending on the slope of the continuous plot of p_det_ against, but this is less commonly used. In practice, it is likely that some patients will be genuinely ‘equivocal’ because obstruction develops gradually.

Schäfer proposed an alternative analysis with the aid of a computer to fit the voiding data to the equations described above.[20] He emphasized the importance of using the parts of the voiding cycle when the outlet is fully relaxed and described his two parameter fit as the ‘passive urethral resistance relationship’ (PURR). He went on to describe a simpler version of this which was based on p_det.Qmax_ and Q_max_, with the addition of an estimate of the urethral opening pressure pura.open. This information was plotted on his ‘linearized passive urethral resistance relationship’ (LPURR) nomogram [Figure 7b].[28] This nomogram indicates several levels of obstruction and also indicates strength of bladder contraction.

An alternative nomogram was proposed by Derek Griffiths and colleagues on the basis that opening pressure is statistically related to the degree of constriction. By plotting p_det.Qmax_ and Q_max_, an effective opening pressure could be interpolated providing a measure of outlet obstruction (43 cmH_2_O in Figure 7c).[29] This has been less widely adopted.

There has been considerable debate about the relative merits of these nomograms. At first sight, they appear quite different but they are based on the same theoretical framework and on closer examination, it can be seen that the boundary separating obstructed patients is identical on the AG nomogram (a) and the Schäfer nomogram (b). The ICS committee making recommendations for interpretation of pressure-flow studies recognized this in their standardization report which introduced the ICS nomogram and uses the same criteria for separating obstructed patients [Figure 7d].[30] The line separating equivocal and unobstructed patients on the ICS nomogram is a compromise of the AG and Schaffer nomograms.

## ADVANTAGES AND DISADVANTAGES OF INVASIVE MEASUREMENTS

### Clinical need

The clinical value of objective measurements in the management of men with LUTS is not universally accepted. Although some urologists will perform prostatectomy without even having a flow rate measurement to support their clinical diagnosis most will measure flow rate as part of their work up before recommending surgery. More controversial is the value of performing an invasive pressure-flow study. Although the literature clearly demonstrates these help diagnose obstruction,[31] some urologists argue the expense, trauma and risk of infection associated with the test are not justified by the modest improvement in the chance of a good outcome from surgery.[32]

### The potential value of a non-invasive alternative

The availability of a non-invasive technique to estimate bladder pressure during voiding and provide a clear urodynamic diagnosis of bladder outlet obstruction without the disadvantages of an invasive study would potentially help resolve this debate. More informed surgery could be performed on patients who are clearly obstructed, and there could be better selection of patients who would benefit from a full pressure-flow study.

## DEVELOPMENT OF NON-INVASIVE URODYNAMICS

There has been a growth of interest in non-invasive urodynamic techniques in recent years in an attempt to maximize diagnostic information before resorting to the invasive test with its attendant costs and risks. The following paragraphs introduce some of these techniques including the ones which are described in this symposium.

### The drop spectrometer

**Figure 8:**
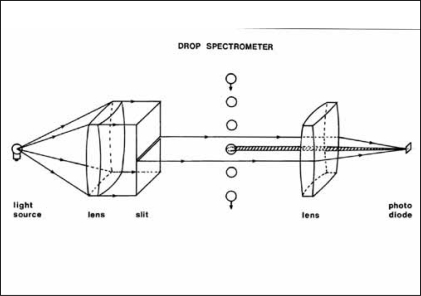
Principle of drop spectrometer. Collimated light passes through a narrow horizontal slit and is refocused onto a photodiode detector. The detected light depends on the size of shadow made by the drop passing through the beam.

This was an interesting development pioneered in the 1960s and 1970s to examine urine flow in more detail. High speed cinematography demonstrates that the urine stream breaks up into drops soon after leaving the external meatus.[33] In the drop spectrometer, the stream is directed though a flat horizontal beam of collimated light aimed towards a photo detector [Figure 8]. The size of the shadows is detected electronically, allowing the timing and size of each drop to be characterized.[34] The technique could detect meatal obstruction but proved less effective at detecting obstruction proximal to the bladder. However, with much greater processing power now available, at low cost, the technique may yet warrant further investigation.[35]

### Condom catheter

**Figure 9:**
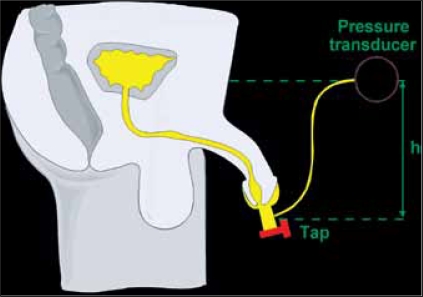
The basic principle of ‘condom catheter’ technique. Flow is interrupted by closing the tap and the pressure builds up. When fluid is stationary, the filled urethra acts as a catheter allowing bladder pressure to be measured.

Schaffer described this technique in the literature, in abstract form,[36] but most of the scientific investigation of the method has been carried out and published by the research group in Rotterdam.[37,38] In principle, a penile drainage sheath is fitted to the penis and a valve used to control the outlet [Figure 9]. With voiding underway, the valve is closed and the pressure builds up. When any compliance in the urethra and condom has been taken up, and flow from the bladder has ceased, the pressure at the valve should equal bladder pressure (allowing for height difference); this pressure is measured via a side port. The Rotterdam group has persevered with the technique, with modifications, and describes their work in this special issue.

### Penile cuff deflation

Gleason and co-workers introduced the interesting idea of applying the principle of non-invasive blood pressure measurement to assess voiding pressure.[39,40] They fitted a paediatric blood pressure cuff to the penis, inflated it and then, while the patient was trying to void, slowly deflated the cuff until flow began.

### Penile cuff inflation

**Figure 10:**
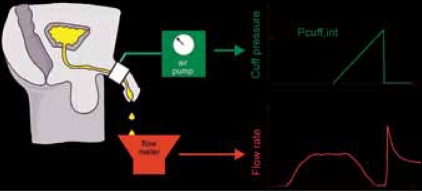
Principle of cuff inflation technique. After voiding has commenced, the cuff is inflated steadily until flow is interrupted. The cuff interruption pressure (p_cuff.int_) is equal to the bladder pressure (plus height difference) when fluid in the urethra is stationary, on the basis of the same principle as non-invasive blood pressure measurement.

Leading on from this the group in Newcastle developed an alternative technique based on inflating the penile cuff at a controlled rate after voiding was underway[41,42] [Figure 10].

This technique is described in a subsequent article in this symposium. We subsequently learned that Brindley and Craggs had published an abstract in 1975 which included the use of a water filled cuff to assess bladder pressure during voiding in man and baboons.[43] Their main research interests lay elsewhere and they did not develop the idea further.

### Penile compression and release

Yalla and colleagues developed a technique in which, during voiding, the subject is asked to grip the penis and interrupt flow for about 3 seconds. On release, the urine stored within the compliant segment of the urethra creates a surge of flow. They compared the magnitude of the surge with subsequent steady flow rate and found this was a good predictor of outlet obstruction.[44] The cuff inflation technique also demonstrates a similar surge of flow when the cuff is released after interruption allowing penile compression ratio (PCR) to be calculated.[45]

### Doppler ultrasound

Doppler ultrasound had been considered unsuitable for urine flow in the urethra because of the lack of scattering properties of urine, demonstrated by the contents of the bladder appearing black on ultrasound images. However, Ozawa *et al.* demonstrated that for flow rates >2 ml/s, turbulence causes cavitation enabling visualization of the stream and hence a Doppler estimation of velocity.[46] In a small group of patients, good agreement was found between invasive classification of obstruction and cross sectional area of the prostatic urethra (calculated from velocity and flow rate) and also with the ratio of velocities in the prostatic urethra and the membranous urethra.[47] The technique requires further validation[48] and the preferred imaging method (transperineally using a robotic arm with the patient seated) requires complex equipment that is not readily available. The principle of the method is described in this symposium.

### Perineal sound

The Rotterdam group also has been using the tendency for the urine stream to become turbulent as it passes through a prostatic obstruction to assess the degree of obstruction by recording acoustic sound via the perineum. Most of the work to date has been based on investigation of a model urethra made from PVA cryogel.[49] Their results with the model show differences in acoustic spectra related to the degree of obstruction (created using a penile cuff). The technique and initial trials in man are described in a subsequent article in this symposium.

### Prostate size and geometry

Intuitively, increased residual volume and prostate size would be expected to relate to outlet obstruction. Results of assessing these measurements have been disappointing, showing only poor correlation.[50] However, more recent studies using more detailed analysis of prostate shape have been more encouraging but further studies are required.[48]

### Bladder wall thickness

In animal experiments where outlet obstruction was deliberately introduced it is known the bladder wall thickens over a period of time.[51–53] This has encouraged the use of ultrasonically measured bladder weight[54] and wall thickness[55] to predict obstruction in men. Oelke and colleagues found that measurement of the anterior detrusor wall thickness (DWT) in an individual using transabdominal imaging was almost independent of bladder volume above 250 ml.[56] The measurement gave good prediction of outlet obstruction in 160 men with a much better ROC curve in comparison to flow rate, residual volume and prostate volume.[57] Provided suitable imaging equipment is available the measurement can be performed quickly but images are open to misinterpretation. The intra and interobserver repeatability of the technique remain to be assessed but in experienced hands this technique may prove to be a valuable alternative non-invasive method for assessing outlet obstruction.[58] Because the measurement methods of DWT and the non-invasive pressure measurements (cuff inflation and condom catheter) are very different and probably independent with respect to extraneous sources of variability, it may prove valuable to combine their results in the assessment of outlet obstruction using, for example, a Bayesian approach.[59]

## SUMMARY

This article has summarized the current state of urodynamic investigation of bladder outlet obstruction including the ‘gold standard’ of invasive pressure-flow studies and the current trend towards the development of non-invasive alternatives. At present, we are in the situation that the most widely accepted and validated method of diagnosis, the invasive pressure flow study, is commonly not performed for practical reasons, but non-invasive techniques are increasingly providing viable alternatives. Several of these techniques await further validation but it is good to see from the work described in subsequent articles that significant progress is being made to this end.
